# Stabilitätsgefährdende Osteolyse der Tibia durch einen intraossären tenosynovialen Riesenzelltumor: Eine außergewöhnliche klinisch-histopathologische Konstellation

**DOI:** 10.1007/s00132-020-03936-2

**Published:** 2020-06-25

**Authors:** Sebastian Klingebiel, Sebastian Mühl, Georg Gosheger, Wolfgang Hartmann, Kristian Nikolaus Schneider, Tymoteusz Borys Budny, Carolin Rickert, Dominik Schorn, Niklas Deventer, Timo Lübben

**Affiliations:** 1 Klinik für Allgemeine Orthopädie und Tumororthopädie, Universitätsklinikum Münster, Albert-Schweitzer Campus 1, 48149 Münster, Deutschland; 2 Gerhard-Domagk-Institut für Pathologie, Universitätsklinikum Münster, Münster, Deutschland

**Keywords:** Amputationsstumpf, Osteosynthese, Osteolyse, PVNS, Verbundosteosynthese, Amputation stump, Composite osteosynthesis, Exoprosthesis, Osteolysis, TSGCT

## Abstract

Der tenosynoviale Riesenzelltumor (TSGCT) ist eine seltene gutartige Erkrankung von Gelenkschleimhäuten, Sehnenscheiden und Schleimbeuteln. Wir berichten über die ungewöhnliche Konstellation einer intraossären Manifestation der proximalen Tibia eines Unterschenkelstumpfes nach Burgess-Amputation infolge eines lokal nicht beherrschbaren tenosynovialen Riesenzelltumors des oberen Sprunggelenkes. Die Kürettage des Lokalbefundes und die operative Stabilisierung durch eine intramedulläre Verbundosteosynthese führten zu einer frühzeitigen Rehabilitation der Exoprothesenversorgung mit Wiedererlangung der Patientenautonomie.

**Video online:**

Die Online-Version dieses Beitrags (10.1007/s00132-020-03936-2) enthält ein Video zur Demonstration des Gangbildes mit angelegter Exoprothese. Beitrag und Video stehen Ihnen auf www.springermedizin.de zur Verfügung. Bitte geben Sie dort den Beitragstitel in die Suche ein, das Zusatzmaterial finden Sie beim Beitrag unter „Ergänzende Inhalte“.

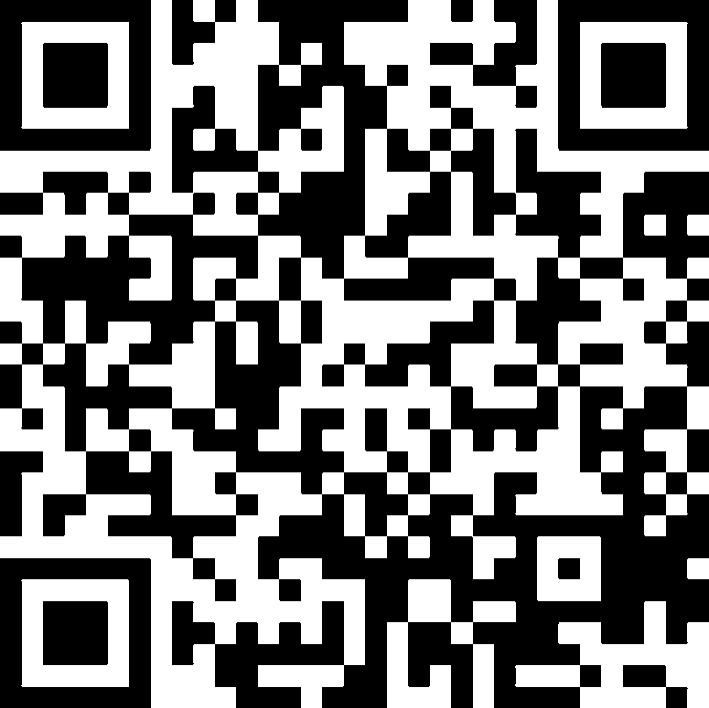

## Abstract (en)

The tenosynovial giant cell tumor is a rare disease of the joint mucosa, tendon sheaths and bursa. We report on the rare constellation of an intraosseous manifestation of the proximal tibia of a lower leg stump after Burgess amputation as a result of a locally uncontrollable tenosynovial giant cell tumor of the upper ankle. The curettage of the local findings and operative stabilization through an intramedullary composite osteosynthesis led to an early rehabilitation of the exoprosthesis care with regaining patient autonomy.

Wir berichten über den seltenen klinischen Fall eines unterschenkelamputierten Patienten mit stabilitätsgefährdender Osteolyse der proximalen Tibia durch eine intraossäre Rezidivmanifestation seitens eines tenosynovialen Riesenzelltumors (TSGCT; vormals: pigmentierte villonoduläre Synovitis). Die Unterschenkelamputation wurde infolge eines lokal nicht beherrschbaren tenosynovialen Riesenzelltumors des oberen Sprunggelenkes durchgeführt. Die intraossäre Manifestation eines TSGCT stellt in diesem Fall eine absolute Rarität und Besonderheit dar.

## Anamnese

**Figure 1:**
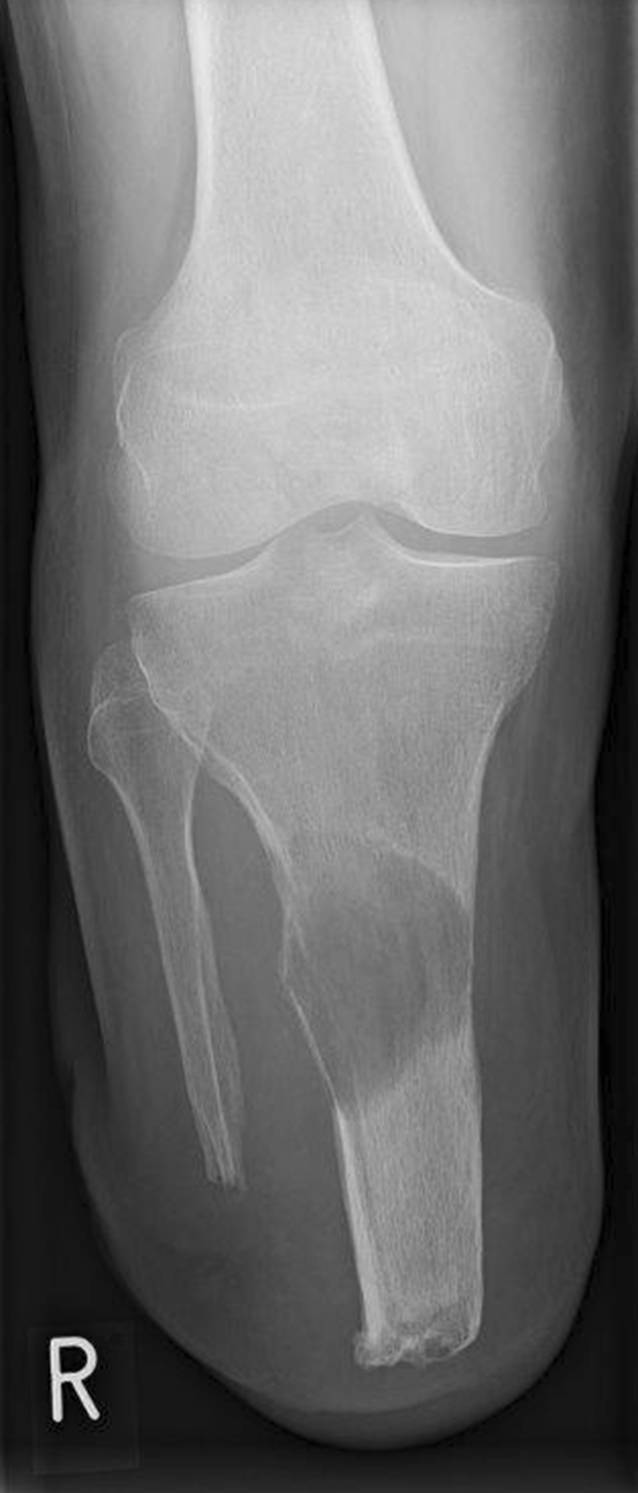

Ein 65-jähriger unterschenkelamputierter Patient stellte sich mit einer großen und stabilitätsgefährdenden Osteolyse der proximalen Tibia seines Unterschenkelstumpfes in unserer Sprechstunde vor (Abb. 1). Er wurde 14 Jahre zuvor aufgrund eines lokal nicht beherrschbaren und rezidivierenden tenosynovialen Riesenzelltumors (TSGCT) des rechten oberen Sprunggelenkes aufgrund exulzerierender extraartikulärer Beteiligung oberhalb der Malleolengabel nach multiplen operativen Revisionen mit einer radikalen Resektion i. S. einer Unterschenkelamputation nach Burgess versorgt. Entsprechend lag damals postoperativ ein R0-Resektionstatus vor. Aufgrund aktuell zunehmender Belastungsschmerzen des Stumpfes in den vergangenen Monaten wurde das bisher komplikationslose Tragen seiner Exoprothese nicht mehr toleriert und der Patient beklagte die hieraus resultierende Immobilität und den drohenden Verlust seiner Autonomie.

## Klinischer und radiologischer Befund

**Figure 2:**
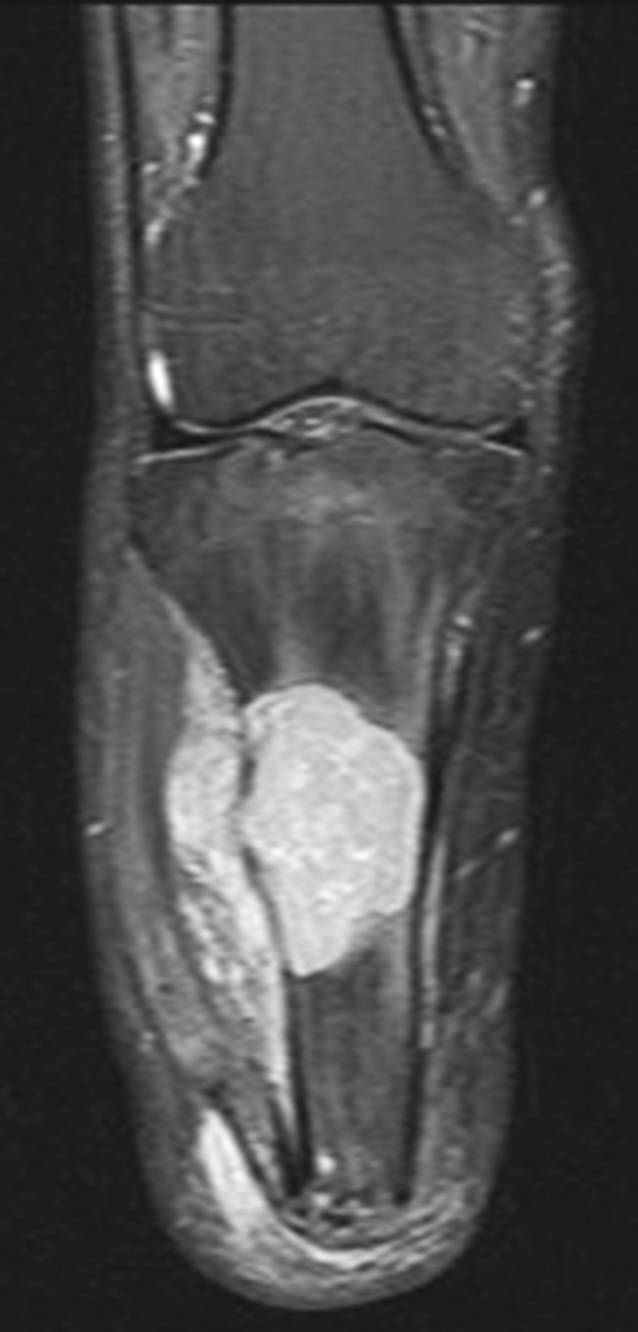

Im Rahmen der klinischen Untersuchung zeigte sich ein äußerlich reizloser Unterschenkelstumpf ohne Infektstigmata oder palpable Resistenzen. Das Kniegelenk war stabil und frei beweglich. Es bestand ein Druckschmerz über der Facies lateralis der Tibia im mittleren Stumpfdrittel. Auf der bereits vorliegenden nativradiologischen Aufnahme konnte eine große osteolytische Raumforderung der proximalen Tibia mit einer Ausdehnung von etwa 5,5 × 4 cm und teilweise fehlender Respektierung der Kortikalis nachgewiesen werden – entsprechend einem Befund Typ 1C nach Lodwick (Abb. 1). Die nachfolgende MRT-Diagnostik bestätigte den Verdacht einer lokal aggressiven ossären Manifestation mit kleiner extraossärer Weichteilkomponente (Abb. 2).

## Diagnose

**Figure 3:**
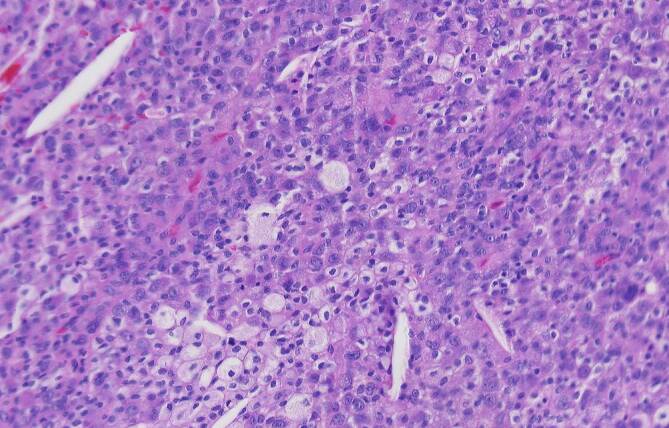

In Zusammenschau stellten wir die dringliche Indikation zur offenen Biopsie. Die konsekutive histopathologische Untersuchung zeigte eine in größeren Teilen CD68- und CD163-positive mononukleäre Proliferation unter Einschluss mehrkerniger Riesenzellen bei Nachweis ausgedehnter Siderinpigmentablagerungen ohne belegbare atypische Mitosen oder Gewebsnekrosen (Abb. 3 und 4). Die Next-Generation-Sequencing-Analyse (NGS) auf DNA-Ebene konnte eine H3F3A-Mutation, wie für einen Riesenzelltumor des Knochens typisch, ausschließen. Mittels RNA-Sequenzierung konnte eine Translokation des CSF1-Genlokus nachgewiesen werden, wie sie für tenosynoviale Riesenzelltumoren bekannt ist. Unter Berücksichtigung der Gesamtkonstellation wurde die Läsion als intraossäre Manifestation eines tenosynovialen Riesenzelltumors vom diffusen Subtyp eingeordnet.

**Figure 4:**
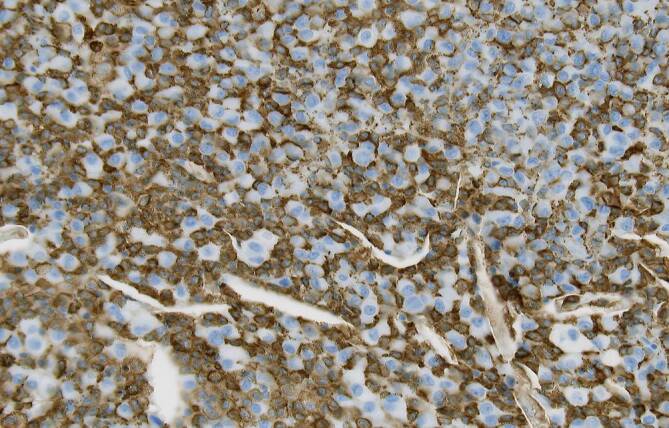

## Therapie und Verlauf

Neben der Indikation zur marginalen Resektion mittels Kürettage diskutierten wir verschiedene Verfahren zur Stabilisierung der proximalen Tibia. Wir entschieden uns zur Durchführung einer intramedullären Verbundosteosynthese mit zwei kanülierten 6,5 × 125 mm Schrauben (Qwix, Fa. Depuy Synthes, Warsaw, IN, USA) und PMMA-Knochenzement (Palacos, Fa. Heraeus medical, Hanau, Deutschland).

**Figure 5:**
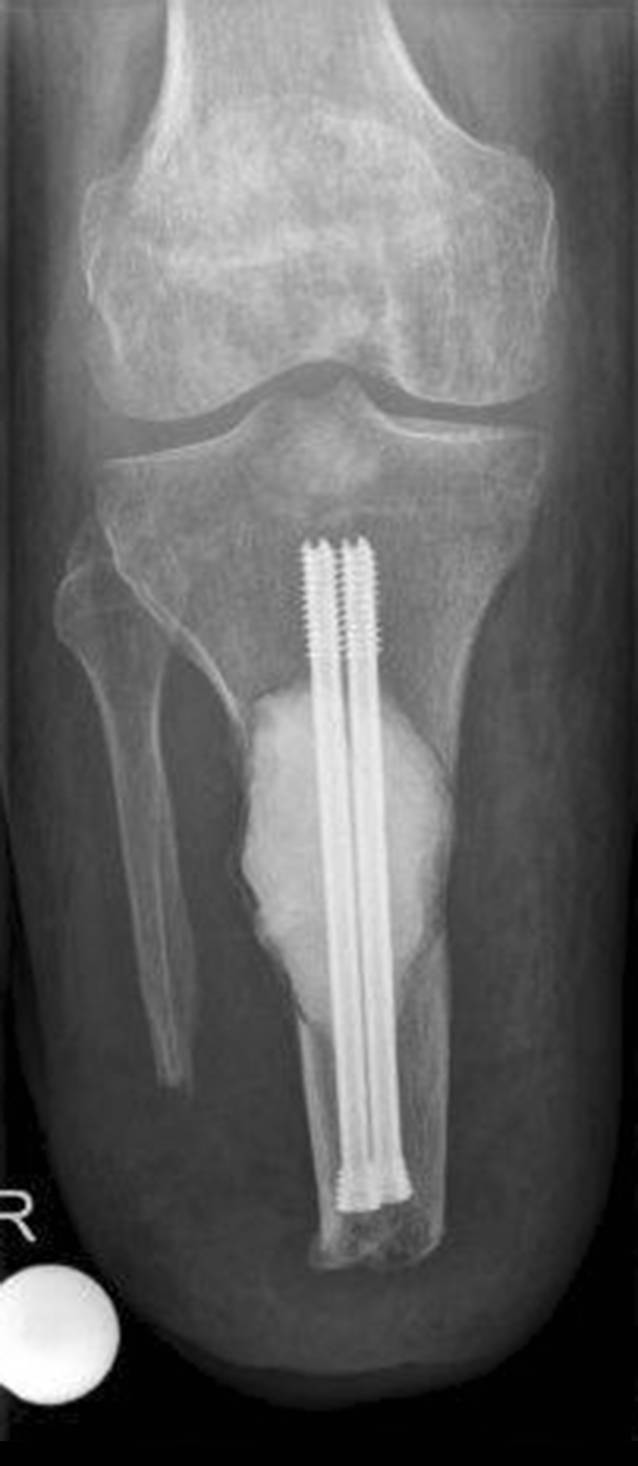

Die Wunde heilte per primam, sodass der Patient am 10. postoperativen Tag bereits erstmals unter Anlage seiner Exoprothese für einige Schritte schmerzfrei mobilisierbar war. Die postoperative Röntgenkontrolle zeigte eine suffiziente Kürettage des TSGCT mit regelrechter Lage der Verbundosteosynthese (Abb. 5). Die postoperative histopathologische Untersuchung des Resektates bestätigte die o. g. Diagnose bei R1-Resektionsstatus.

**Figure 6:**
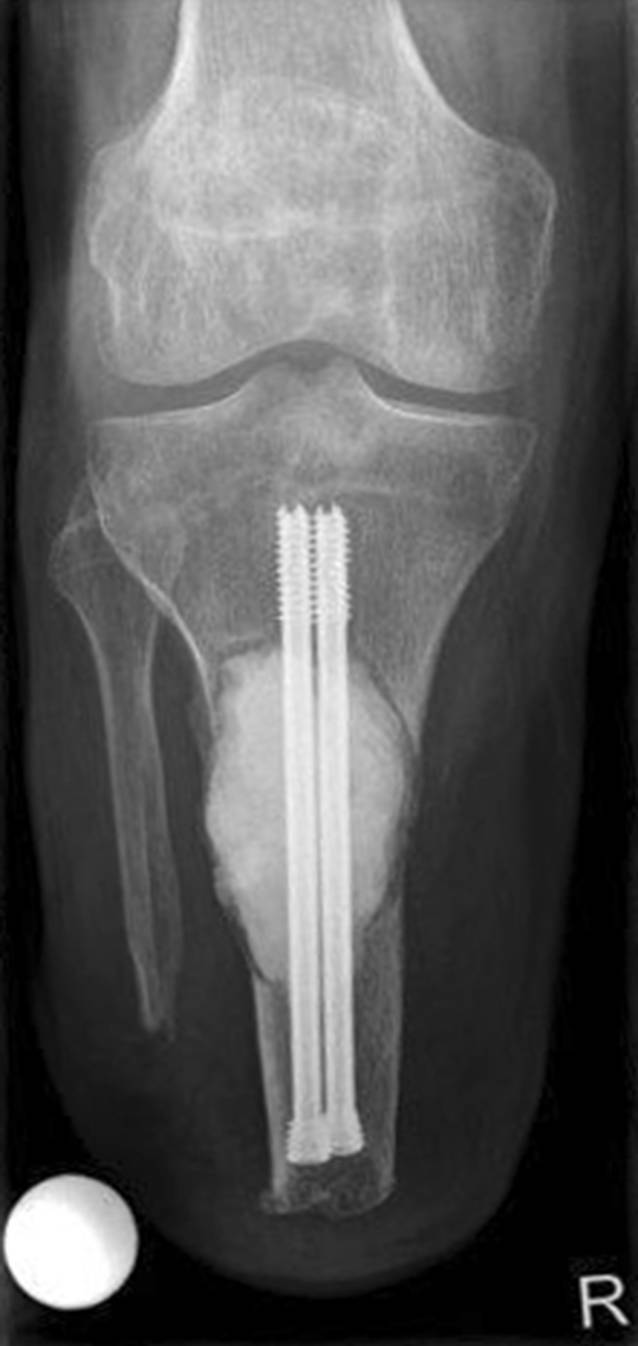

**Figure 7:**
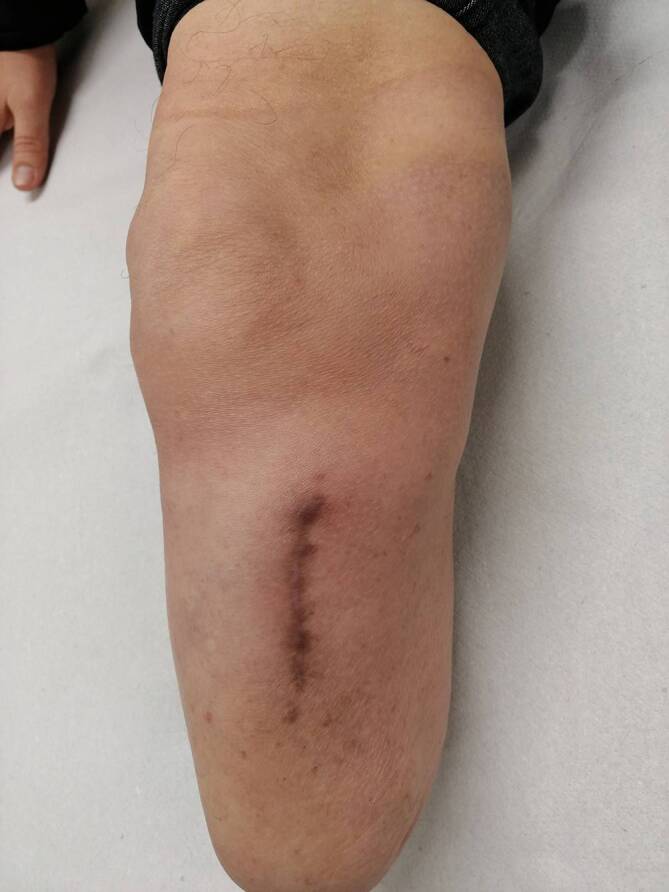

**Figure 8:**
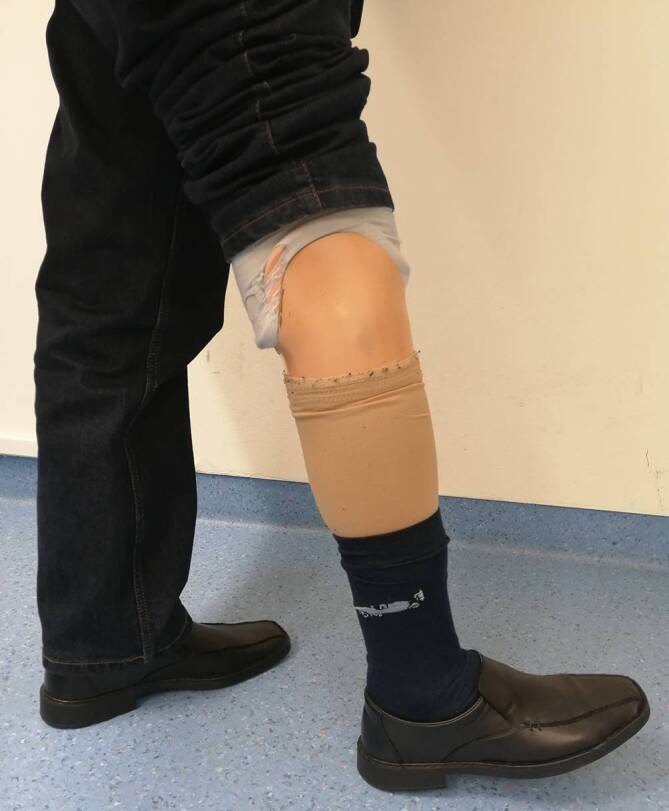

Eine planmäßige Wiedervorstellung zur klinisch-radiologischen Verlaufskontrolle erfolgte 3 Monate postoperativ. Nativradiologisch und MR-graphisch zeigte sich ein konstanter Befund ohne Hinweis auf ein lokales Tumorrezidiv (Abb. 6). Eine pulmonale Manifestation wurde aufgrund der aggressiven Biologie des hier beschriebenen TSGCT als Einzelfallentscheidung CT-graphisch ausgeschlossen. Der Patient berichtete im Rahmen dieser Konsultation bereits über eine vollständige Remobilisation mit unlimitierter Gehstrecke. Er konnte seinen Alltag wieder ohne Einschränkungen bewältigen. Die Narbe war reizlos verheilt und der Stumpf regelrecht konfiguriert (Abb. 7). Der Patient demonstrierte mit angelegter Exoprothese (Abb. 8) ein flüssiges und weitestgehend physiologisches Gangbild (siehe Video online zu diesem Beitrag).

Wir haben dem Patienten aufgrund des relativ hohen Risikos für ein Lokalrezidiv weiterhin engmaschige klinisch-radiologische Verlaufskontrollen im 3‑monatigen Intervall für zunächst 2 Jahre postoperativ mit lokaler kontrastmittelgestützter MRT-Bildgebung und Projektionsradiographie empfohlen. Anschließend sollten bis einschließlich des 5. postoperativen Jahrs halbjährliche Kontrollen erfolgen.

## Diskussion

Der TSGCT ist mit einer geschätzten Inzidenz von 1,5–2/1.000.000 eine seltene gutartige, aber potenziell lokal aggressive Erkrankung der Gelenkschleimhaut des überwiegend jungen Patienten zwischen 20 und 40 Jahren [7]. Der klinisch-ätiologische Hintergrund von TSGCT ist weitestgehend unklar. Einige Autoren sehen gar in über 50 % der Fälle einen Zusammenhang mit traumatischen Ereignissen [6]. Andere Autoren sehen eine inflammatorische Genese vordergründig [1]. Es wird unterschieden zwischen der lokalen und der diffusen Form des TSGCT. Histologisch sind diese Typen nicht zu unterscheiden. Erstere Form ist klinisch gut umschrieben und kann sich intra- sowie extraartikulär manifestieren. Insbesondere die diffuse Form des TSGCT kann sich lokal aggressiv und mit infiltrativem Wachstumsmuster ausbreiten und manifestiert sich klinisch in erster Linie durch eine schmerzhafte Gelenkschwellung [9, 11]. Genetisch konnte in TSGCT als rekurrentes Ereignis in einer Subpopulation von Zellen eine Translokation mit Beteiligung des CSF1-Genlokus nachgewiesen werden, die über eine CSF1-Überexpression zur Rekrutierung von Makrophagen führt [10]. Prädilektionsstellen dieser auch durch Hämosiderineinlagerungen charakterisierten, zumeist synovialen Läsionen sind große Gelenke wie Knie‑, Sprung- und Hüftgelenk (v. a. diffuser Typ), aber auch Sehnenscheiden und Schleimbeutel (eher lokaler Typ) [6]. Trotz sorgfältiger arthroskopischer oder offener Resektion sind Lokalrezidive keine Seltenheit [2]. Bei der diffusen Form sind Rezidivraten von bis zu 40 % beschrieben [5]. Daher stellen TSGCT eine besondere diagnostische und therapeutische Herausforderung in der Orthopädie und Rheumatologie dar. Schwerwiegende Verläufe mit lokal nicht beherrschbarem Wachstum und maligner Transformation oder ungewöhnlichen Lokalisationen wie dem Temporomandibulargelenk sind Ausnahmen, jedoch in der Literatur beschrieben [4, 8]. Der hier präsentierte Fall, bei dem etliche Jahre zuvor eine lokale Tumorkontrolle des TSGCT nur durch eine radikale Resektion im Sinne einer Unterschenkelamputation mit R0-Status herbeigeführt werden konnte, stellt eine absolute Rarität dar. Eine exzeptionelle Konstellation ist bei dieser Rezidivmanifestation die nahezu ausschließlich intraossäre Lokalisation des TSGCT. Die Manifestation des TSGCT im Knochen, offenbar als Sekundärmanifestation, zeigt in diesem Einzelfall das für einen TSGCT ungewöhnliche aggressive biologische Potenzial des Prozesses. Dass dieser Befund dennoch kein Einzelfall ist, zeigen aktuelle Fallberichte mit Beschreibung eines TSGCT im Os sacrum und Entwicklung neurologischer Symptomatik, sowie ein weiterer Fall mit Infiltration von Azetabulum und Oberschenkelhals bei TSGCT des Hüftgelenkes [3]. Dass es sich in unserem Fallbeispiel bei dem TSGCT des Amputationsstumpfes um ein Spätrezidiv handelt, ist zwar wahrscheinlich, schlussendlich jedoch nicht sicher nachvollziehbar. Die zeitliche Latenz von 14 Jahren bis zur Manifestation eines sehr späten Rezidivs und das offensichtlich in diesem Fall diskontinuierliche Wachstum sind hierbei eine weitere außergewöhnliche Konstellation. Bei der Literaturrecherche ließ sich eine vergleichbare Konstellation nach R0-Reektion nicht identifizieren.

Das erfreuliche postoperative Ergebnis der oben dargestellten unkonventionellen Verbundosteosynthese, die dem Patienten die Wiedererlangung seiner Mobilität gewährte, ist in diesem Fall ebenfalls hervorzuheben. Weitere engmaschige klinisch-radiologische Verlaufskontrollen werden zeigen, ob das bisherige klinische Ergebnis bestätigt werden kann. Durch die Art der Versorgung mit der intramedullären Verbundosteosynthese konnte zum einen das Kniegelenk als funktionell entscheidendes Element der suffizienten und bisher seitens des Patienten gut angenommenen Exoprothesenversorgung erhalten werden. Die Verwendung des PMMA-Zementes hat neben der Augmentation der Läsion auch den Aspekt der Adjuvanz im Sinne einer Thermodesinfektion im Rahmen der exothermen Reaktion bei Aushärtung. Dies kann der lokalen Tumorkontrolle dienen. Zum anderen konnte hierdurch eine auftragende Plattenosteosynthese der proximalen Tibia unterbleiben. Diese hätte möglicherweise für Druckschäden im Weichgewebe oder Irritationen der Liner- und Prothesenanpassung gesorgt, sodass eine dauerhafte adäquate Exoprothesenversorgung gefährdet gewesen wäre. In der Literatur konnte eine vergleichbare Stumpfversorgung nicht gefunden werden, wodurch der experimentelle Charakter dieser operativen Therapie unterstrichen wird.

## Fazit für die Praxis

- Ein TSGCT (tenosynovialer Riesenzelltumor) kann sich grundsätzlich intraossär als osteolytische Läsion manifestieren. Hierbei handelt es sich jedoch um eine absolute Rarität.
- Die marginale Resektion (R1-Status) eines TSGCT ist bei klinischer Symptomatik der therapeutische Goldstandard und führt in der Regel zu einer lokalen Tumorkontrolle. Dennoch sind Lokalrezidive vor allem beim diffusen Typ des TSGCT häufig.
- Die experimentelle intramedulläre Verbundosteosynthese der proximalen Tibia mit zwei kanülierten Schrauben und PMMA(Polymethylmethacrylat)-Knochenzement führte bei einem unterschenkelamputierten Patienten mit intraossärem TSGCT zur vollständigen Rehabilitation mit uneingeschränkter Exoprothesenversorgung und Wiedererlangung seiner Autonomie.
- Die PMMA-Augmentierung in diesem Fall weist neben dem Vorteil der vereinfachten nativradiologischen Rezidivdiagnostik möglicherweise einen positiven Effekt auf die lokale Tumorkontrolle durch Thermodesinfektion auf.

## Caption Electronic Supplementary Material



## Abkürzungen

CD: „Cluster of differentiation“ (immunphänotypische Oberflächenmerkmale von Zellen)
CSF: „Colony stimulating factor“
HE: Hämatoxylin-Eosin (Färbeverfahren in der Histologie)
PMMA: Polymethylmethacrylat (Synthetischer Knochenzement)
STIR: „Short tau inversion recovery“ (MRT-Sequenz)
TSGCT: Tenosynovialer Riesenzelltumor
